# Naloxone Administration Frequency During Emergency Medical Service Events — United States, 2012–2016

**DOI:** 10.15585/mmwr.mm6731a2

**Published:** 2018-08-10

**Authors:** Rebecca E. Cash, Jeremiah Kinsman, Remle P. Crowe, Madison K. Rivard, Mark Faul, Ashish R. Panchal

**Affiliations:** ^1^National Registry of Emergency Medical Technicians, Columbus, Ohio; ^2^Office of Emergency Medical Services, National Highway Traffic Safety Administration, Washington, D.C.; ^3^Association of Schools and Programs of Public Health, Washington, D.C.; ^4^Division of Unintentional Injury Prevention, National Center for Injury Prevention and Control, CDC; ^5^Department of Emergency Medicine, The Ohio State University Wexner Medical Center, Columbus, Ohio.

As the opioid epidemic in the United States has continued since the early 2000s (*1*,*2*), most descriptions have focused on misuse and deaths. Increased cooperation with state and local partners has enabled more rapid and comprehensive surveillance of nonfatal opioid overdoses (*3*).* Naloxone administrations obtained from emergency medical services (EMS) patient care records have served as a useful proxy for overdose surveillance in individual communities and might be a previously unused data source to describe the opioid epidemic, including fatal and nonfatal events, on a national level (*4*–*6*). Using data from the National Emergency Medical Services Information System (NEMSIS),^†^ the trend in rate of EMS naloxone administration events from 2012 to 2016 was compared with opioid overdose mortality rates from National Vital Statistics System multiple cause-of-death mortality files. During 2012–2016, the rate of EMS naloxone administration events increased 75.1%, from 573.6 to 1004.4 administrations per 100,000 EMS events, mirroring the 79.7% increase in opioid overdose mortality from 7.4 deaths per 100,000 persons to 13.3. A bimodal age distribution of patients receiving naloxone from EMS parallels a similar age distribution of deaths, with persons aged 25–34 years and 45–54 years most affected. However, an accurate estimate of the complete injury burden of the opioid epidemic requires assessing nonfatal overdoses in addition to deaths. Evaluating and monitoring nonfatal overdose events via the novel approach of using EMS data might assist in the development of timely interventions to address the evolving opioid crisis.

NEMSIS Public Release Research data sets from 2012 through 2016 were used for this analysis. Approximately 10,000 EMS agencies and 49 U.S. states and territories contribute data to the NEMSIS National EMS Database, resulting in a national convenience sample of EMS events (*7*). EMS naloxone administration events were defined as the administration of at least 1 naloxone dose during EMS patient care. EMS events for this evaluation included 9-1-1 responses, responses during special event coverage, and provision of care by EMS crew in an ambulance intercept^§^ or during mutual aid to another ambulance response.^¶^ Those events in which opioid analgesics were administered by EMS, where no patient was found by the responding EMS crew, or where the event was a medical transport or interfacility transfer were excluded. Because the focus of this evaluation was on rates of naloxone administration events as a proxy to opioid overdoses, rather than severity of overdoses, multiple naloxone dosing was not considered. Administration of naloxone by EMS is the standard of care for many EMS systems in the prehospital setting for patients in cardiac arrest and those who are unconscious. Thus, recognizing that not all naloxone administrations by EMS represent actual opioid overdoses, a subanalysis of suspected overdoses, defined as the subset of EMS events with naloxone administration and documented evidence of drug ingestion/poisoning,** was conducted to obtain the potential range of actual opioid overdoses treated by EMS. The primary outcome examined was the annual rate of naloxone administration per 100,000 EMS events with a secondary analysis of trends in patient characteristics. Chi-squared tests of linear trend were used to compare data across the 5 yearly time points (2012 to 2016) along with the percent increase over this period.

The estimated rate of EMS naloxone administration was compared with opioid overdose mortality rates reported in CDC’s National Vital Statistics System multiple cause-of-death mortality files during 2012–2016.^††^ Following the methodology used in past work used to describe drug overdose mortality (*2*), opioid-involved deaths during the study period, with underlying causes of death related to poisoning with a multiple cause of death involving opioids, were queried by year.^§§^

From 2012 to 2016, a stepwise increase occurred in the number of EMS events with naloxone administration (Table 1). The increase persisted in the subset of suspected overdoses, EMS naloxone administrations with documented evidence of a drug ingestion/poisoning (Table 2). During 2012–2016, the rate of naloxone administration events overall increased 75.1%, from 573.6 to 1,004.4 administrations per 100,000 EMS events (Table 2), and the rate of naloxone administration in suspected overdoses increased 119.0%, from 230.6 to 505.2. Concomitant with the increase in naloxone administration rates was a 79.7% increase in age-adjusted opioid mortality rate, from 7.4 deaths per 100,000 persons in 2012 to 13.3 in 2016 (Table 2).

**TABLE 1:** Patient demographics for emergency medical services (EMS) event records with documented administration of naloxone — United States, 2012– 2016

Characteristic	Year, no. (%)					P–value*
	2012 (N = 91,853)	2013 (N = 108,957)	2014 (N = 123,400)	2015 (N = 167,182)	2016 (N = 207,584)	
**Suspected overdose^†^**	36,933 (40.2)	45,002 (41.3)	53,601 (43.4)	79,611 (47.6)	104,412 (50.3)	<0.001
**Age group (yrs)**						
0–14	628 (0.7)	605 (0.6)	718 (0.6)	859 (0.5)	1,080 (0.5)	<0.001
15–24	11,715 (12.8)	13,159 (12.1)	14,350 (11.7)	19,759 (11.9)	23,135 (11.2)	
25–34	15,686 (17.2)	18,955 (17.5)	22,947 (18.7)	35,179 (21.2)	47,411 (23.0)	
35–44	13,910 (15.2)	16,190 (14.9)	18,325 (14.9)	25,929 (15.6)	33,979 (16.5)	
45–54	18,049 (19.8)	20,815 (19.2)	22,812 (18.6)	29,491 (17.7)	36,333 (17.6)	
55–64	14,014 (15.3)	17,557 (16.2)	19,930 (16.2)	26,366 (15.9)	32,439 (15.7)	
65–74	7,808 (8.5)	9,856 (9.1)	11,380 (9.3)	14,271 (8.6)	16,431 (8.0)	
≥75	9,575 (10.5)	11,341 (10.5)	12,344 (10.1)	14,463 (8.7)	15,684 (7.6)	
**Male**	49,343 (54.0)	59,492 (54.9)	69,564 (56.7)	97,542 (58.6)	126,600 (61.3)	<0.001
**Race**						
White	57,438 (78.0)	65,786 (76.2)	73,257 (75.6)	96,625 (75.0)	112,277 (72.0)	<0.001
Black	11,062 (15.0)	14,639 (17.0)	17,018 (17.6)	23,660 (18.4)	33,338 (21.4)	
Other^§^	5,182 (7.0)	5,871 (6.8)	6,680 (6.9)	8,618 (6.7)	10,370 (6.7)	

**Source:** National Emergency Medical Services Information System (https://nemsis.org/), 2012–2016.

* Nonparametric test of trend.

^†^ EMS records were included if any of the following were documented as drug ingestion, poisoning, or overdose: complaint reported by dispatch (E03_01, field value 510), EMS provider’s primary impression (E09_15, field value 1690), EMS provider’s secondary impression (E09_16, field value 1825), and cause of injury (E10_01, field value 9530).

^§^ American Indian or Alaska native, Asian, Native Hawaiian or other Pacific Islander, other/unknown.

**TABLE 2:** Rates of emergency medical services (EMS) naloxone administration events and opioid overdose deaths — National EMS Information System (NEMSIS) and CDC National Vital Statistics System, United States, 2012–2016*

Year	NEMSIS^†^ EMS naloxone administration events rate (95% CI)		CDC^¶^ opioid-involved death rate (95% CI)
	Overall	Suspected opioid^§^	
2012	573.6 (569.9–577.3)	230.6 (228.3–233.0)	7.4 (7.3–7.5)
2013	666.0 (662.0–669.9)	275.1 (272.5–277.6)	7.9 (7.8–8.0)
2014	691.3 (687.4–695.1)	300.3 (297.7–302.8)	9.0 (8.9–9.1)
2015	805.1 (801.3–809.0)	383.4 (380.7–386.1)	10.4 (10.3–10.5)
2016	1,004.4 (1,000.1–1,008.7)	505.2 (502.1–508.3)	13.3 (13.2–13.4)
% Change**	75.1	119.0	79.7

**Abbreviation:** CI = confidence interval.

* Naloxone administration event rate expressed as rate per 100,000 EMS events; age-adjusted mortality rate expressed per 100,000 persons.

^†^ Per 100,000 EMS events. Data from NEMSIS (https://nemsis.org/), 2012–2016.

^§^ EMS records were included if any of the following were documented as drug ingestion, poisoning, or overdose: complaint reported by dispatch (E03_01, field value 510), EMS provider’s primary impression (E09_15, field value 1690), EMS provider’s secondary impression (E09_16, field value 1825), and cause of injury (E10_01, field value 9530).

^¶^ Per 100,000 population. Data from CDC’s National Vital Statistics System, Multiple Cause of Death Data, 2012–2016; CDC WONDER (https://wonder.cdc.gov). To obtain estimates of opioid-involved deaths from the Multiple Cause of Death Data see https://wonder.cdc.gov; *International Classification of Disease, Tenth Revision* (ICD-10) codes X40–X44, X60–X64, X85, and Y10–Y14 were used for underlying cause of death and ICD-10 codes T40.0, T40.1, T40.2, T40.3, T40.4, and T40.6 were used for multiple cause of death.

** Percent change calculated from 2012 to 2016.

A bimodal distribution was observed in the age groups of patients who received naloxone during EMS events, with modes at ages 25–34 years and 45–54 years (Table 1) (Figure). In 2012, a larger proportion of naloxone administration events occurred among persons aged 45–54 years (19.8%, 18,049) than among persons aged 25–34 years (17.2%, 15,686, p<0.001). By 2016, this finding had reversed, and a larger proportion of naloxone administration events occurred among persons aged 25–34 years (21.2%, 35,179) than among persons aged 45–54 years (17.7%, 29,491, p<0.001). A similar bimodal age distribution was also identified in opioid overdose deaths from 2012 to 2016, mirroring the two modes observed in EMS data (Figure).

**FIGURE:**
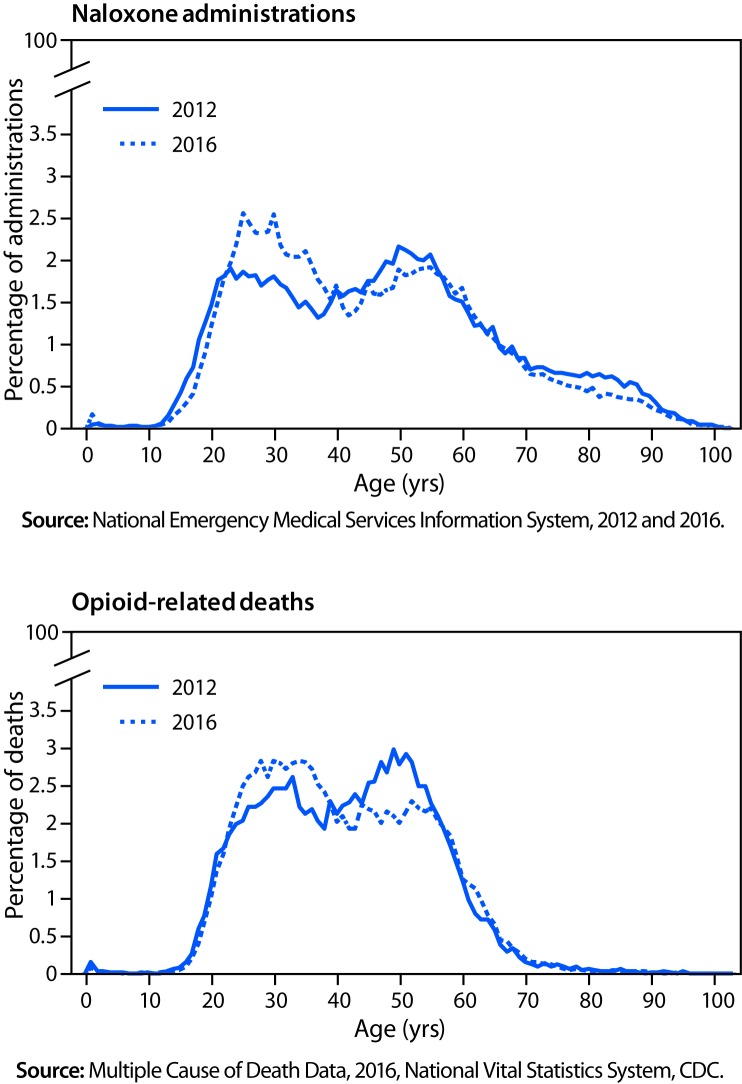
Percentage of naloxone administrations by emergency medical services and percentage of opioid-related deaths, by age — United States, 2012 and 2016

## Discussion

This cross-sectional evaluation of the large NEMSIS Public Release Research data sets from 2012 to 2016 demonstrated that the increase in the rate of all naloxone administration by EMS parallels the increase in rate of fatal opioid overdoses. As proposed, examining naloxone administrations by EMS professionals might be a useful and timely tool to gauge the comprehensive prevalence of opioid overdoses, including those that do not end in a fatal event. EMS data regarding naloxone administration can be used by health care organizations and communities to benchmark the performance of interventions over time and compare with national averages, as well as assist in the development of timely interventions.

This analysis also demonstrated a bimodal age distribution in both naloxone administrations by EMS and opioid overdose deaths. Further, a trend of increasing naloxone administrations and deaths in younger persons (aged 25–34 years) was observed. The reasons for these findings are difficult to discern from these data. Whereas efforts have been increased to control access to and misuse of prescription opioid pain relievers, use of illicit opioids such as fentanyl is increasing (*8*,*9*). Use of heroin and illicitly manufactured fentanyl is associated with younger age groups (*9*,*10*). This change from misuse of prescription opioid pain relievers to highly potent illicit opioids offers a plausible explanation for the increased prevalence of naloxone administration by EMS and deaths in the younger age group. However, rates of drug overdose deaths have increased in all age groups, with convergence of these rates for those aged 25–34, 35–44, and 45–54 years.^¶¶^ As this prevalence of disease changes, there is a potential impact on years of life lost caused by opioid overdose in the United States.

The novel use of EMS naloxone administration data to examine nonfatal overdoses, in conjunction with mortality and emergency department data (*3*), provides a more robust picture of the burden of injury for opioid overdose epidemic. The overall rate of naloxone administration increased by 75.1% from 2012 to 2016. In the subgroup analysis of suspected overdose events, the increase was even higher (119.0%), suggesting that EMS providers are increasingly more likely to administer naloxone in borderline cases. Because these patients represent a population still at risk for overdose and death, more work is needed to understand nonfatal overdose events.

The findings in this report are subject to at least five limitations. First, this analysis focused on naloxone administered by EMS personnel in a large convenience sample of EMS records. The accuracy and completeness of data entered into the NEMSIS Public Release Research data set are dependent on correct and thorough entries by EMS personnel. The accuracy of these data are unknown, limiting the ability to assess the rate of naloxone use by laypersons and non-EMS personnel. Second, naloxone use by laypersons or other first responders, including law enforcement, without activation of the EMS system is not reflected in these data sets. Third, variations in EMS record documentation submitted to the NEMSIS National EMS Database might present a potential misclassification bias. Fourth, because these data were deidentified, it was not possible to assess naloxone administration over repeated events. Although the increased use of potent illicit opioids has resulted in multiple naloxone administrations during many EMS events (*6*), multiple naloxone administrations and dosages of naloxone given by EMS were not assessed. Finally, because this was a secondary analysis of cross-sectional data, causality cannot be inferred.

Evaluating and monitoring nonfatal overdose events might assist in the development of more timely emergency response interventions, more naloxone administrations in suspected drug overdose cases, and referral to treatment and care coordination. EMS agencies and their communities can also compare naloxone administrations with national benchmarks to evaluate the effectiveness of interventions. EMS data are useful for identifying populations at risk, such as those surviving an opioid overdose, and could assist in meeting the challenge of decreasing the mortality impact of the opioid epidemic. Further, these results support widening the scope of discussion concerning opioid epidemic overdoses and demonstrate the importance of EMS providers in providing a more complete evaluation of opioid overdose injury burden in the United States.

**Summary**

**What is already known about this topic?**

Naloxone administration data from emergency medical services (EMS) records have been used for surveillance for opioid overdoses on a local level.

**What is added by this report?**

Analysis of a national database of EMS events found that from 2012 to 2016, the rate of naloxone administrations increased 75.1%, from 573.6 to 1004.4 per 100,000 EMS events, mirroring a 79.7% increase in the age-adjusted opioid mortality rate.

**What are the implications for public health practice?**

Monitoring nonfatal overdose events using EMS records provides a more complete evaluation of the potential injury burden and a means of benchmarking for communities and EMS agencies to better address the evolving opioid epidemic.

## Footnotes

* https://www.cdc.gov/drugoverdose/foa/state-opioid-mm.html.

† https://nemsis.org/.

§ Ambulance intercepts occur when one EMS provider meets a transporting EMS unit with the intent of receiving a patient or providing a higher level of care. https://nemsis.org/media/nemsis_v2/documents/Data_Managers_Council_-_Data_Definitions_Project_Final_Ve..pdf.

** EMS records were included if any of the following were documented as drug ingestion, poisoning, or overdose: “complaint reported by dispatch (E03_01, field value = 510),” “EMS provider’s primary impression (E09_15, 1690),” “EMS provider’s secondary impression (E09_16, 1825),” and “cause of injury (E10_01, 9530).”

†† https://wonder.cdc.gov/.

§§ To obtain estimates of opioid-involved deaths from the Multiple Cause of Death Data (https://wonder.cdc.gov), the *International Classification of Disease, Tenth Revision* (ICD-10) codes of X40–X44, X60–X64, X85, and Y10–Y14 were used for underlying cause of death and ICD-10 codes of T40.0, T40.1, T40.2, T40.3, T40.4, and T40.6 were used for multiple cause of death.

¶¶ https://www.cdc.gov/nchs/products/databriefs/db294.htm.

## References

[1] Mack KA, Jones CM, Ballesteros MF. Illicit drug use, illicit drug use disorders, and drug overdose deaths in metropolitan and nonmetropolitan areas—United States. MMWR Surveill Summ 2017;66(No. SS-19). 10.15585/mmwr.ss6619a1 29049278 PMC5829955

[2] Rudd RA, Seth P, David F, Scholl L. Increases in drug and opioid-involved overdose deaths—United States, 2010–2015. MMWR Morb Mortal Wkly Rep 2016;65:1445–52. 10.15585/mmwr.mm655051e1 28033313

[3] Vivolo-Kantor AM, Seth P, Gladden RM, Vital signs: trends in emergency department visits for suspected opioid overdoses—United States, July 2016–September 2017. MMWR Morb Mortal Wkly Rep 2018;67:279–85. 10.15585/mmwr.mm6709e1 29518069 PMC5844282

[4] Knowlton A, Weir BW, Hazzard F, EMS runs for suspected opioid overdose: implications for surveillance and prevention. Prehosp Emerg Care 2013;17:317–29. 10.3109/10903127.2013.792888 23734988 PMC3682796

[5] Lindstrom HA, Clemency BM, Snyder R, Consiglio JD, May PR, Moscati RM. Prehospital naloxone administration as a public health surveillance tool: a retrospective validation study. Prehosp Disaster Med 2015;30:385–9. 10.1017/S1049023X15004793 26061280

[6] Faul M, Lurie P, Kinsman JM, Dailey MW, Crabaugh C, Sasser SM. Multiple naloxone administrations among emergency medical service providers is increasing. Prehosp Emerg Care 2017;21:411–9. 10.1080/10903127.2017.1315203 28481656 PMC6026856

[7] Mann NC, Kane L, Dai M, Jacobson K. Description of the 2012 NEMSIS public-release research dataset. Prehosp Emerg Care 2015;19:232–40. 10.3109/10903127.2014.959219 25290075

[8] Compton WM, Jones CM, Baldwin GT. Relationship between nonmedical prescription-opioid use and heroin use. N Engl J Med 2016;374:154–63. 10.1056/NEJMra1508490 26760086 PMC11784537

[9] O’Donnell JK, Halpin J, Mattson CL, Goldberger BA, Gladden RM. Deaths involving fentanyl, fentanyl analogs, and u-47700—10 states, July–December 2016. MMWR Morb Mortal Wkly Rep 2017;66:1197–202. 10.15585/mmwr.mm6643e1 29095804 PMC5689219

[10] Jones CM, Logan J, Gladden RM, Bohm MK. Vital signs: demographic and substance use trends among heroin users—United States, 2002–2013. MMWR Morb Mortal Wkly Rep 2015;64:719–25. 26158353 PMC4584844

